# *Sarocladium spinificis*, a new endophytic species from the coastal grass *Spinifex littoreus* in Taiwan

**DOI:** 10.1186/1999-3110-55-25

**Published:** 2014-02-05

**Authors:** Yu-Hung Yeh, Roland Kirschner

**Affiliations:** Department of Life Sciences, National Central University, No. 300, Jhongda Road, Jhongli City, Taoyuan County 32001, (R.O.C.) Taiwan

**Keywords:** Ascomycota, Coastal dunes, Endophytes, Sandy beach, Taxonomy

## Abstract

**Background:**

*Sarocladium* species are frequently associated with grasses as saprobes, parasites, and mutualistic endophytes. A species of *Sarocladium* (anamorphic Hypocreales) was isolated as endophytic fungus from the coastal grass *Spinifex littoreus* (Poaceae).

**Results:**

According to characterization by LSU and ITS rDNA sequences and culture morphology and micromorphology, the species differed from the species hitherto described in *Sarocladium*. A key to the known species of *Sarocladium* is given.

**Conclusions:**

*Sarocladium spinificis* is proposed as a new species. LSU rDNA sequences and conidiophore branching and conidium size are useful characters for distinguishing between species of *Sarocladium*.

**Electronic supplementary material:**

The online version of this article (doi:10.1186/1999-3110-55-25) contains supplementary material, which is available to authorized users.

## Background

The genus *Sarocladium* was erected by Gams and Hawksworth (1976) based on a species described by Sawada in Taiwan, first including two fungal pathogens causing sheath rot of rice, with the type species *Sarocladium oryzae* (Sawada) W. Gams & D. Hawksw., and *S. attenuatum* W. Gams & D. Hawksw. (Gams and Hawksworth, 1976). The characteristics of the genus are phialides being narrowly cylindrical, hardly tapering towards the apices, lacking collarettes, producing one-celled, cylindrical, hyaline conidia (Gams and Hawksworth, 1976). *Sarocladium sinense* J.D. Chen, Guo C. Zhang & X.H. Fu and *S. mycophilum* Helfer were described in 1987 and 1991, respectively (Helfer, 1991; Liao and Wu, 2000). In 2011, according to phylogenetic analysis based on sequences of the LSU rDNA, *Sarocladium* was extended to include 7 species formerly placed in *Acremonium* (Summerbell et al., 2011). By molecular analysis, *Sarocladium* can be connected to the Hypocreales, but the relationship with a teleomorph on the species, genus, and family level is completely unknown. The species of *Sarocladium* have many important applications. For example, *S. oryzae* produces antibiotics, *S. kiliense* (Grütz) Summerb. can cause human diseases, *S. strictum* is able to cause disease of sorghum and strawberry plants, and *S. zeae* (W. Gams & D.R. Sumner) Summerb. is considered a mutualistic maize endophyte protective against herbivory (Tschen et al., 1997; Bills et al., 2004; Summerbell et al., 2011; Racedo et al., 2013, as *Acremonium strictum*). Most strains have been isolated from members of Poaceae, such as bamboo, maize, rice, and other cereals and wild grasses (Gams, 1971; Gams and Hawksworth, 1976; Summerbell et al., 2011).

On the coast of Taiwan, the grass *Spinifex littoreus* (Burm. f.) Merr. (Poaceae) grows in littoral sandy beaches and dunes. To propagate and over-come sand burial, the species produces vertical and horizontal rhizomes and stolons. By this growth, populations of this plant contribute to the formation of dunes (Maun, 2009). Though *Sp. littoreus* is important for stabilizing and protecting coastal areas, very little has been published about the organisms associated with this plant. For this reason, we studied endophytes and other fungi associated with this plant. Among the fungi most frequently isolated as endophytes, a species was identified as member of *Sarocladium*, but was not identical to any of the previously described species.

## Methods

### Plants and collection sites

During the years 2011–2012, individuals of *Spinifex littoreus* were collected in seven locations distributed on sandy sites of the northern, western and southern coast of Taiwan. Plants were collected from five beaches of five counties in Taiwan (Yilan 24.718°N, 121.832°E, Taoyuan 25.047°N, 121.076°E, Hsinchu 24.765°N, 120.911°E, Miaoli 24.622°N, 120.756°E and Taipei 25.029°N, 121.935°E), while in the two remaining counties (Pingtung and Chiayi), plants were obtained at other habitats. In Pingtung County, plants were collected at Hengchun Tropical Botanic Garden (21.958°N, 120.811°E), 200–300 m above sea level, at a distance of 2 km from the coast. In Chiayi, the site was coastal wetland close to Haomeiliao (23.360°N, 120.129°E). The plants were processed for endophyte isolation within 72 hours after sampling.

### Isolation of fungi

Plants were removed with a trowel, individually placed in bags, returned to the laboratory and kept at 4°C until further processing. Samples were washed and divided into root, stem, leaf sheath and leaf lamina. All healthy plant parts from *Spinifex littoreus* were cut transversally into three fragments. Furthermore, seeds of *Sp. littoreus* were used. Plant fragments were surface-sterilized by agitation in 95% ethanol for 1 min, 6–12% sodium hypochlorite for 3 min, 95% ethanol for 0.5 min, and then rinsed in sterile water, and placed onto malt extract agar (MEA). The effectiveness of surface sterilization was tested by making imprints of plant fragments on malt extract agar plates (Rodriguez et al., 2008). All isolates obtained from each plant sample were classified according to their morphological appearance into morphotypes. Representative isolates of a species of *Sarocladium* were investigated and deposited at the Bioresource and Collection Center, Hsinchu, Taiwan (BCRC). A dried culture was deposited at the herbarium of the National Museum of Natural Science, Taichung, Taiwan (TNM).

### DNA isolation and molecular analysis

**Figure 1:**
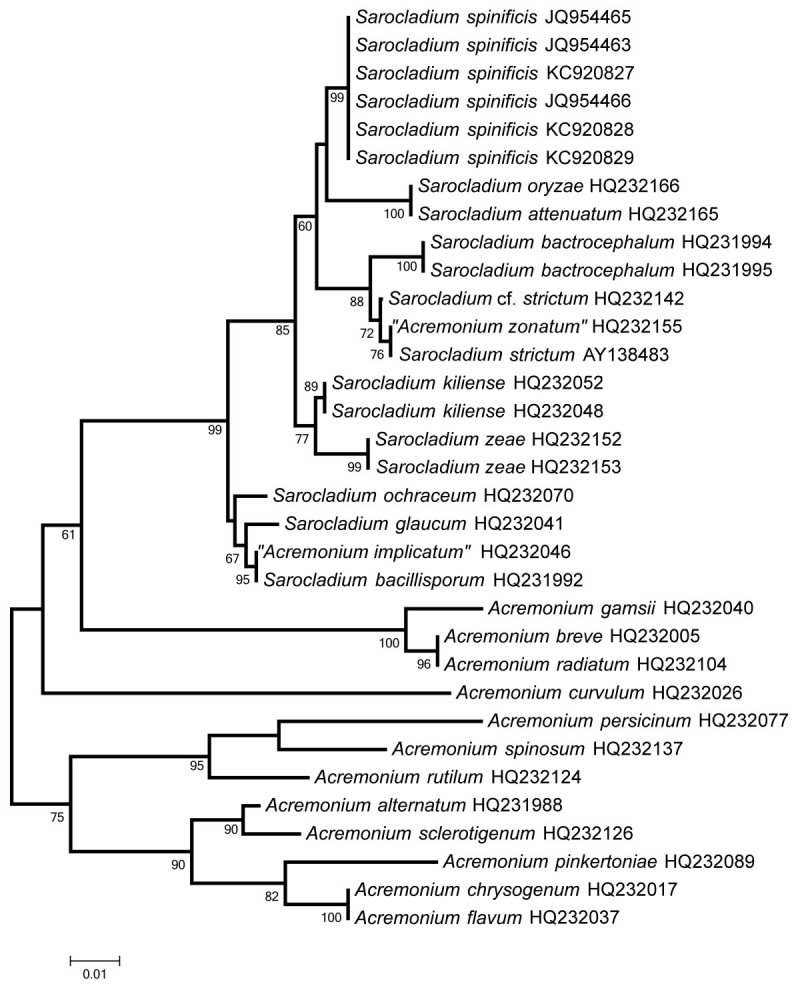
**Phylogenetic tree derived from analysis of the LSU rDNA sequences of species of** ***Sarocladium*** **and related** ***Acremonium*** **species using the Maximum Likelihood method by MEGA5.** Bootstrap support values above 50% of 1000 replications given below the branches.

DNA was extracted from all examined strains with Genomic DNA Spin Kit (Plant), according to the modified manufacturer’s protocol (Bioman Scientific Co., Ltd., Taiwan). Primer pairs ITS1F/ITS4 and NL1/NL4 were used for amplification of the ITS and partial LSU rDNA, respectively (Gardes and Bruns, 1993; White et al., 1990). Success of the amplification was assessed with 2% agarose gel electrophoresis followed by staining with GelRed™ (Biotium, Hayward, California, U.S.A.) visualized under UV light (312 nm). Illustra GFX PCR DNA and Gel Band Purification Kit, GE Healthcare, UK, were applied for purification of the PCR products. Sequencing of DNA was done by Mission Biotech (Nankang, Taipei) with the same primers as for the PCR. DNA sequences were edited using CodonCode Aligner version 4.0.1 (CodonCode Corporation, USA). Related DNA sequences of ITS and LSU rDNA were compared using the BLAST function of GenBank. For phylogenetic analysis, sequences retrieved from the BLAST search and the taxon sampling based on LSU rDNA sequences in Summerbell et al. (2011) were used. In addition to sequences of *Sarocladium* spp., those of *Acremonium* species closely related with *Sarocladium* spp., but outside the *Sarocladium* clade, were included as outgroup (Summerbell et al., 2011). Altogether 27 nucleotide sequences were aligned using the default options of MUSCLE implemented in MEGA5 (Tamura et al., 2011) without manual editing except for truncating the ends so that a block of 481 positions in the final dataset was obtained. The evolutionary history was inferred by using the Maximum Likelihood method with the default options of MEGA5, based on the Tamura-Nei model and 1000 bootstrap replications. The tree shown in Figure 1 was not rooted.

### Morphological study

Fungal growth rates were evaluated after transfer of conidia with a flamed needle onto three plates containing 2% malt-extract agar (MEA) with 0.2% chloramphenicol and incubation at 25°C. After 10 days, the diameter of the colony was measured. For evaluating the culture morphology, corn meal agar (CMA) and potato dextrose agar (PDA) were also used. Online Auction Color Chart® was used as reference for color terms. Microscopic characteristics were observed using fungal material mounted in 5–10% (w/v) aqueous KOH solution and 1% phloxine. Statistical treatment of measurements was based on 30 measurements of the phialides of the designated ex-type strain which are given as mean value ± standard deviation with extreme values given in brackets. The same method was used for 30 conidia per each of five strains, i.e. altogether 150 conidia.

## Results

Among 1670 isolates, 307 (ca. 18%) were identified as *Sarocladium* sp. An analysis of all endophytes will be presented in another publication. As shown in Figure 1, the isolates from *Spinifex littoreus* (GenBank accession numbers of newly created LSU rDNA sequences JQ954465, JQ954463, KC920827, JQ954466, KC920828, KC920829) form a strongly supported clade which is significantly separated from sequences of other *Sarocladium* species. A sequence of the ITS (594 bp) was deposited in GenBank as KF269096. When comparing the BLAST search results among sequences exceeding a length of 539 bp of ITS rDNA fragments, the highest similarity between our isolates and other identified *Sarocladium* species was 95%, namely with one strain of *Sarocladium strictum* (Genbank number AY428790, Saleh and Leslie, 2004) showing a similarity of 94%–95%. According to morphology, the new species is most similar to *Sarocladium strictum*, because *S. strictum* is a variable species without clear-cut diagnostic characteristics (Domsch et al., 2007).

### Taxonomy

**Figure 2:**
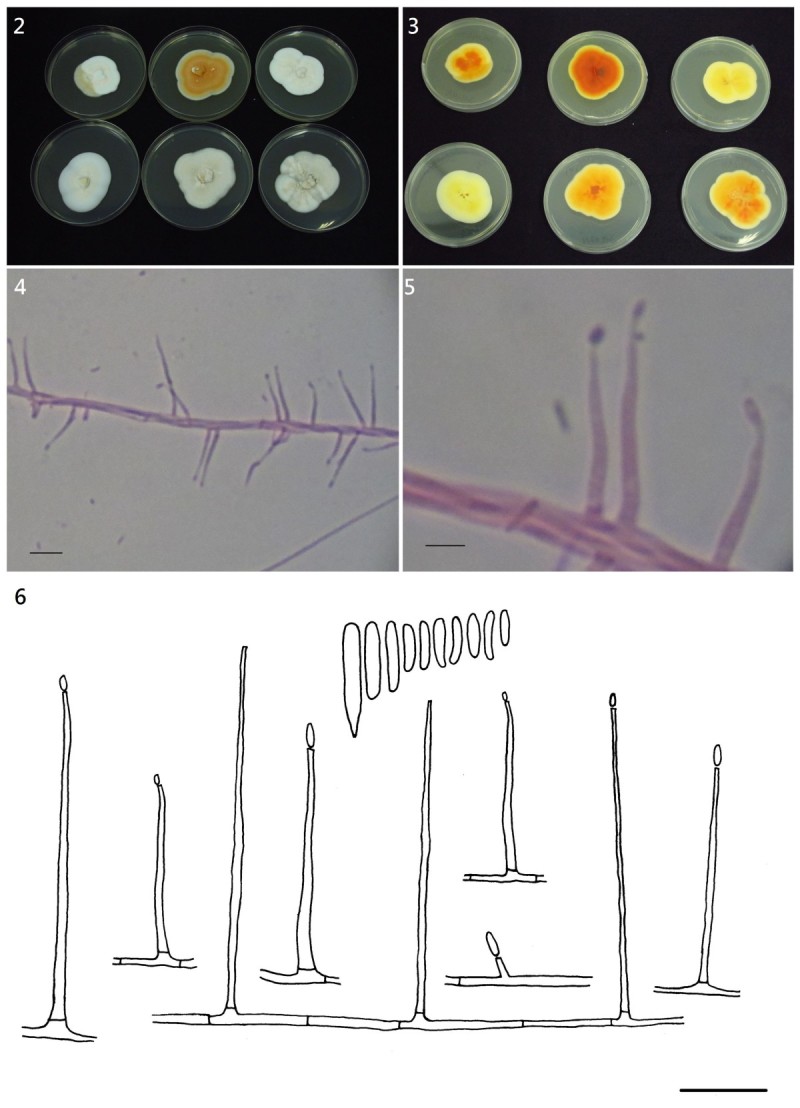
***Sarocladium spinificis*** **. 2–3**, Culture morphology of six strains; colonies on malt extract agar after 10 days at 25°C in the dark (upper isolates from left to right S0101, Z0504, Z1602, lower from left to right Z0106, Z1003, Z1701. 9 cm Petri dishes); **2**, Upper side; **3**, Lower side; **4–5**, Phialides on hyphae (stained with phloxine); **6**, Drawing of phialides and conidia. Scale bars: **4** = 20 μm, **5–6** = 10 μm.

***Sarocladium spinificis*** Yu Hung Yeh & R. Kirschner, sp. nov. Figure 2(2-6).

MycoBank no.: MB 805250.

### Holotypus

Dried culture from isolate from root fragment of living *Spinifex littoreus*, Taiwan, Taoyuan County, Kuanyin Township, 27.04.2011, Yu-Hung Yeh Z0106 (TNM).

Ex-type strain: BCRC 34941. LSU rDNA sequence: GenBank JQ954463.

Colonies on malt extract agar 28.5–30 mm diam. after 10 days at 25°C in the dark, flat or at colony centre folded into low wrinkles, overall mycelium appressed forming conspicuous concentric zones. Surface color white (colors Oac909) to pale orange (colors Oac683 and Oac794), reverse orange yellowish (ranging from colors Oac812 to Oac814), sometimes a soluble yellow pigment diffusing into the agar. On CMA, the color is white, on PDA yellow.

Hyphae smooth, hyaline, 1–3 μm wide, without swellings or chlamydospores. Conidiophores reduced to conidiogenous cells, not stained with cotton blue. Phialides attached singly to subtending hyphae, 20–37.5(-42) × 1.5–2 μm, robust in appearance, mostly straight, subcylindrical, tapering towards the apex up to 0.5–1 μm, with a small, indistinct, collarette. Adelophialides rare, 3 × 1 μm. Conidia formed in slimy heads, cylindrical or ellipsoidal, hyaline, smooth, 5–8(-13) × 1–2 μm (n = 30). Conidial widths of five different strains including the ex-type strain (30 conidia measured per strain) were identical (1–2 μm), but maximum lengths were slightly different, namely 7–9(-13) μm.

### Etymology

Referring to the host genus *Spinifex*.

### Teleomorph

Unknown.

### Other specimens examined with cultures deposited at BCRC

All isolated as endophytes from living *Spinifex littoreus*, Taiwan; Miaoli County, Houlong Township, from root, 01.08.2011, Yu-Hung Yeh Z0504 (BCRC FU30127, LSU rDNA: KF269096; ITS sequence: KF269096); Chiayi County, coastal wetland close to Haomeiliao, from stem, 02.12.2011, Yu-Hung Yeh Z1003 (BCRC FU30128, LSU rDNA: JQ954466); from seed, 02.12.2011, Yu-Hung Yeh S0101 (BCRC FU30126, LSU rDNA: JQ954465); same place, from root, 25.06.2012, Yu-Hung Yeh Z1701 (BCRC FU30130, LSU rDNA: KC920829); Pingtung County, Hengchun Tropical Botanic Garden, 200–300 m above sea level, from stem, 10.06.2012, Yu-Hung Yeh Z1602 (BCRC FU30129, LSU rDNA: KC920828).

## Discussion

The DNA results indicate that the species is to be identified as a member of *Sarocladium*, which is confirmed by morphology. The species is proposed as new because of DNA sequence differences, yellow-orange pigmentation of the cultures, absence of conidiophore branching, and comparatively long conidia. Some strains produce a soluble yellow pigment that diffuses into the agar. After the most recent revision of *Acremonium* and *Sarocladium* (Summerbell et al., 2011) the genus hitherto contains 11 species. DNA data are available for nine species (Summerbell et al., 2011; this report). Additionally, the morphological characters of the 11 described species were compiled from the literature: *S. attenuatum*, *S. bacillisporum* (Onions & G.L. Barron) Summerb., *S. bactrocephalum* (W. Gams) Summerb., *S. glaucum* (W. Gams) Summerb., *S. kiliense*, *S. mycophilum*, *S. ochraceum* (Onions & G.L. Barron) Summerb., *S. oryzae*, *S. sinense*, *S. strictum* (W. Gams) Summerb., and *S. zeae* (Gams and Hawksworth, 1976; Helfer, 1991; Liao and Wu, 2000). None of these species produces a soluble pigment diffusing into the agar. Only six of eleven species form somewhat yellow or orange colonies (*S. bacillisporum*, *S. kiliense*, *S. mycophilum*, *S. ochraceum*, *S. strictum*, and *S. zeae*). Among these six species, branched conidiophores were reported for *S. kiliense*, *S. mycophilum*, *S. strictum* and *S. zeae*, whereas branched conidiophores are absent in the new species. *Sarocladium strictum* is a variable species with few particular characteristics (Domsch et al., 2007). In *S. strictum*, branched conidiophores are usually absent and colonies pink colored, and conidia are shorter than in all the rest of the species (Gams, 1971; Domsch et al., 2007). It can, however, also include sometimes orange colonies and occasionally branched conidiophores (de Hoog et al., 2000). In some strains, both unusual characteristics (orange colonies and branched conidiophores) are combined together. Such untypical strains of *S. strictum* could be distinguished only by shorter conidia [3.3–5.5(-7.0) μm, Gams, 1971 and molecular data from *S. spinificis. Sarocladium bacillisporum* and *S. ochraceum* are the only species with yellow or orange colonies and unbranched conidiophores. *Sarocladium bacillisporum* differs from *S. spinificis* by its catenate conidia. *Sarocladium spinificis* differs from *S. ochraceum* by having longer conidia [5–8(-13) × 1–2 μm vs. 4.4–5.1 × 1.3–1.5 μm]. In *S. ochraceum*, the conidia contribute to the ochre yellow color of dusty dry colonies (Gams, 1971). Summerbell et al. (2011) indicated that *Acremonium implicatum* (J.C. Gilman & E.V. Abbott) W. Gams might belong to *Sarocladium*. This species, however, produces conidia in dry chains (Gams, 1971). For *Acremonium*, Gams species now classified in *Sarocladium* (1971) and Domsch et al. (2007) indicated further similar *Acremonium* species for morphological comparison. From our comparison of *S. spinificis* with the descriptions of these species, we concluded that *S. spinificis* is distinct.

*Sarocladium* now comprises 12 species (if *S. attenuatum* and *S. oryzae* are considered separate species), of which eight species are discussed in Summerbell et al. (2011), including seven species described in detail by Gams (1971), but scattered among different species of *Acremonium*, whereas the descriptions of the other species are given in further publications (Gams and Hawksworth, 1976; Helfer, 1991; Liao and Wu, 2000). We compiled the diagnostic characters of the 12 species from the literature in order to construct following key:

1. Conidiophores almost branched … 2
2. Conidiophores unbranched … 3
3. Culture color yellow, becoming brown from the center, on fungi …

… *S. mycophilum* Helfer

2. Culture color different from above … 4
3. Culture color is intensively greyish green …

… *S. glaucum* (W. Gams) Summerb.

3. Culture color different from above … 8
4. Conidia usually up to 7 μm long, causing rice sheath blast … 5
4. Conidia usually up to 6 μm, not causing disease on rice … 6
5. Conidium size 4.5–8(-14) × 0.6–1.0 μm …

… *S. attenuatum* W. Gams & D. Hawksw.

5. Conidium size 3.5–7 × 1–1.5 μm …

… *S. oryzae* (Sawada) W. Gams & D. Hawksw.

5. Conidium size 3.75–11.5 × 1.75–2.54 μm …

… *S. sinense* J.D. Chen, Guo C. Zhang & X.H. Fu

6. With chlamydospores, colonies on Sabouraud agar brown …

… *S. kiliense* (Grütz) Summerb.

6. Without chlamydospores … 7
7. Conidiophore often repeatedly branched; tips of phialides with localized wall thickening and frequently wavy in outline beneath this, conidiophore base strongly stained with blue stains …

…*S. zeae* (W. Gams & D.R. Sumner) Summerb.

7. Conidiophores usually consisting of phialides; phialides lacking wall thickening and smooth in outline, in some case somewhat stainable with blue stains…

… *S. strictum* (W. Gams) Summerb.

8. Conidia usually up to 8 μm long, on *Spinifex littoreus* … *S. spinificis*
8. Conidia shorter … 9
9. Conidia usually in chains …

… *S. bacillisporum* (Onions & G.L. Barron) Summerb.

9. Conidia in slimy drops … 10
10. Conidia with pointed ends, colonies dry and dusty …

… *S. ochraceum* (Onions & G.L. Barron) Summerb.

10. Not as above … 11
11. Conidia < 1 μm wide … *S. bactrocephalum* (W. Gams) Summerb.
11. Conidia ≥ 1 μm wide … *S. strictum* (W. Gams) Summerb.

Possibly the three species *Sarocladium attenuatum*, *S. oryzae*, and *S. sinense* are the same species. A possible synonymy of *S. attenuatum* and *S. oryzae* was proposed by Bridge et al. (1989) based on biochemical and morphometric analyses and further supported because of identical DNA sequences (Bills et al., 2004). The ITS sequence obtained from a culture of *S. attenuatum*, however, by Summerbell et al. (2011) was different from that obtained from the same culture by Bills et al. (2004). We did not find morphological differences in the description of *S. sinense* by Liao and Wu (2000) from descriptions of *S. oryzae* (Gams and Hawksworth, 1976). These three species all cause sheath disease in *Oryza sativa* and have no significant difference in morphology.

*Sarocladium* spp. are often associated with Poaceae, such as *S. attenuatum*, *S. oryzae*, and *S. sinense* in rice. *Sarocladium zeae* is known as a protective maize endophyte (Wicklow et al., 2005; Poling et al., 2008). *Sarocladium bacillisporum*, *S. bactrocephalum* and *S. strictum* also have been recorded as endophytes from Poaceae (Tunali et al., 2000; Hormazabal and Piontelli, 2009). By molecular techniques, the most abundant clones isolated from hypersaline microbial mats revealed to belong to *S. strictum* (as *Acremonium strictum*, Cantrell et al., 2013), indicating that *Sarocladium* species might have the ability to grow in environments with high salinity. Since *S. spinificis* has been discovered as grass endophyte from a marine coastal habitat, we hypothesize that this species has a similar function as other endophytes conferring salt tolerance to coastal plants (Rodriguez et al., 2008).

## Conclusions

*Sarocladium spinificis* isolated as endophyte from the coastal plant *Spinifex littoreus* is proposed as a new species. LSU rDNA sequences in addition to conidiophore branching and conidium size are useful characters for distinguishing between species of *Sarocladium*.

## Authors’ original submitted files for images

Below are the links to the authors’ original submitted files for images. Authors’ original file for figure 1 Authors’ original file for figure 2
