# Supplementary figures and images for: Sarocladium spinificis, a new endophytic species from the coastal grass Spinifex littoreus in Taiwan
Source: Bot Stud. 2014 Feb 5;55:25. doi: 10.1186/1999-3110-55-25 (PMC5430317; doi:10.1186/1999-3110-55-25)

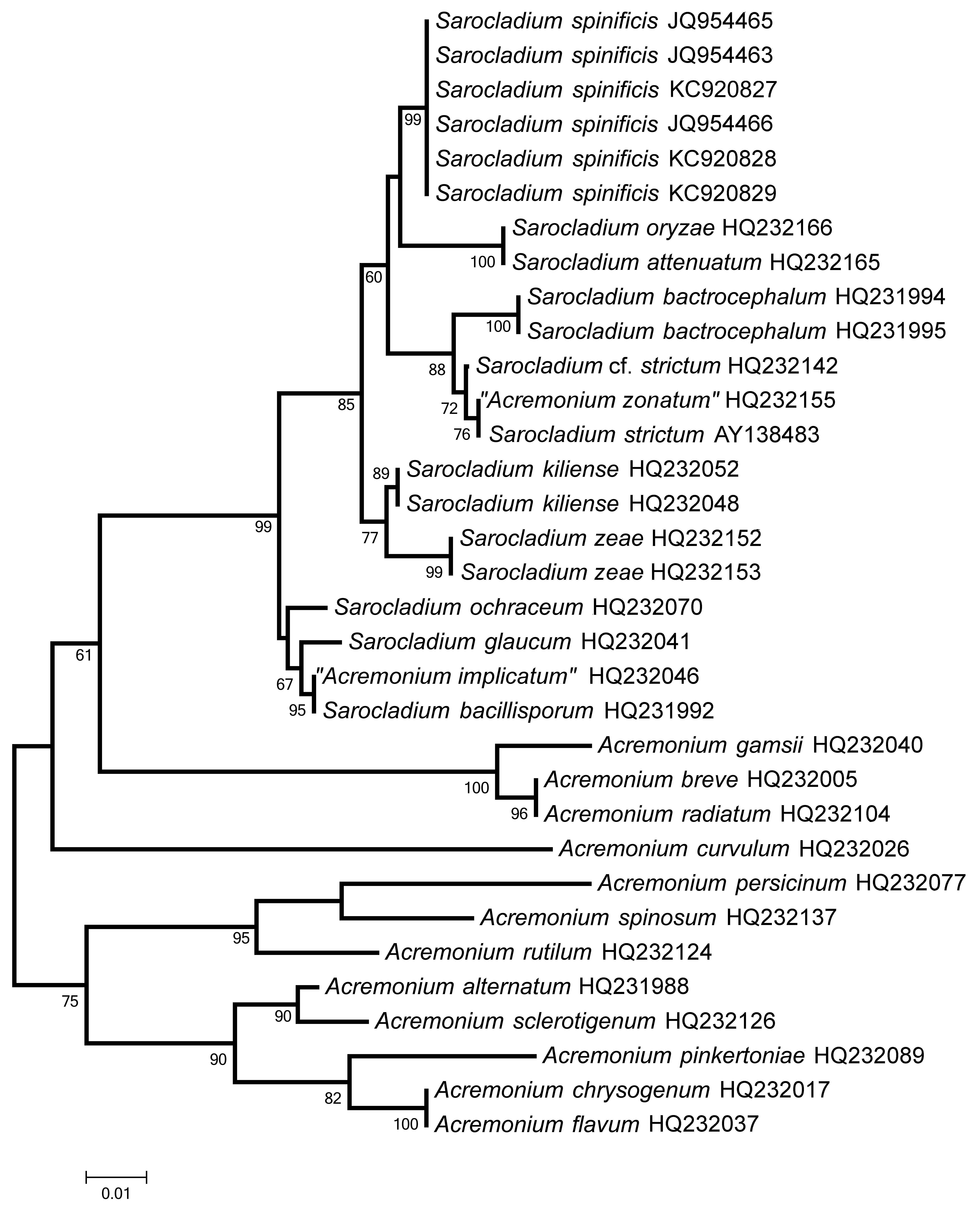

Supplement: Supplementary file 1 — Authors’ original file for figure 1 [file 40529_2013_73_MOESM1_ESM.jpeg]

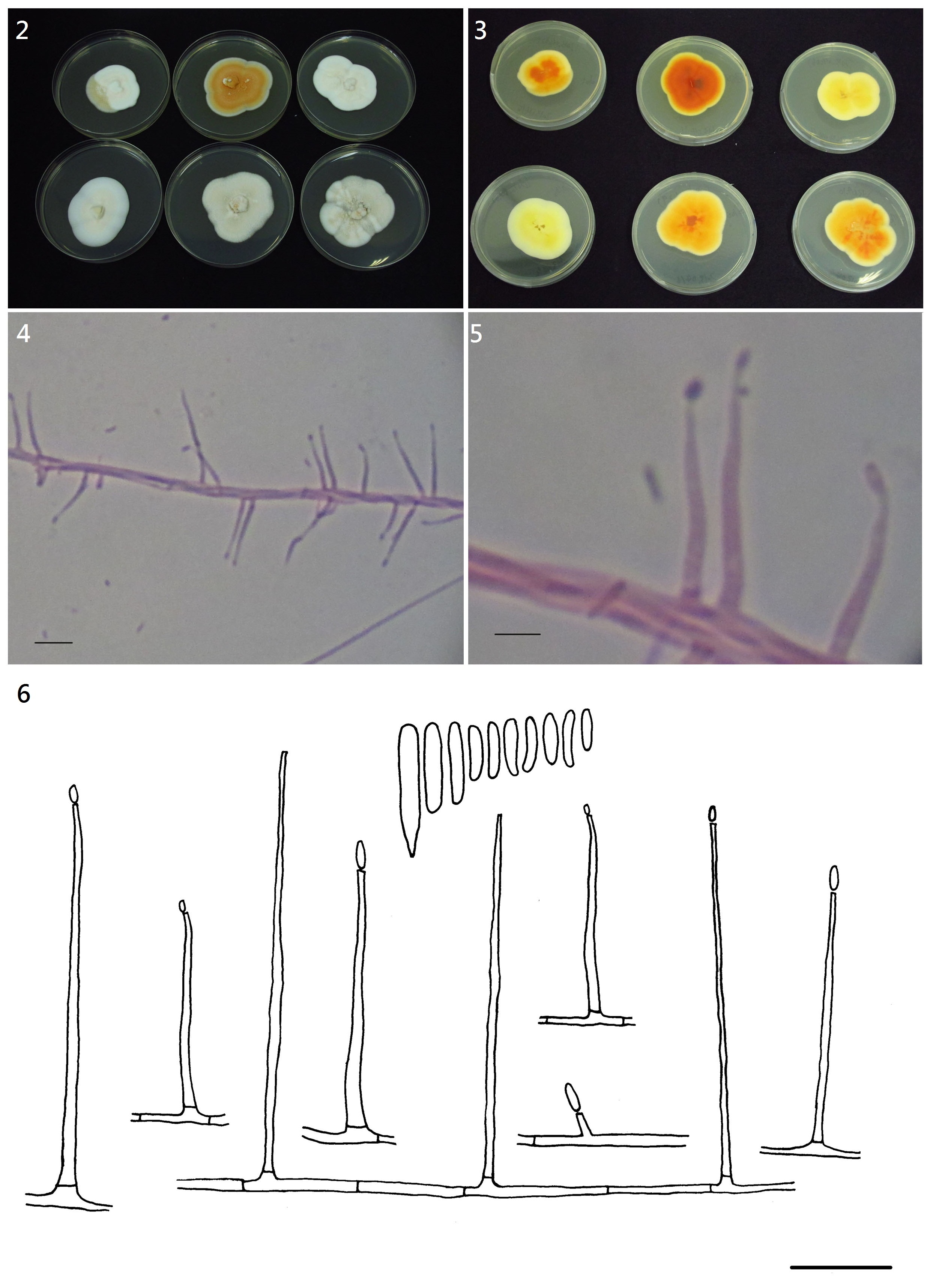

Supplement: Supplementary file 2 — Authors’ original file for figure 2 [file 40529_2013_73_MOESM2_ESM.jpeg]
